# Potential und Herausforderung eines ambulanten Rehabilitationsprogramms für Kinder und Jugendliche mit Adipositas – Interviews mit Fachpersonal aus Rehabilitationseinrichtungen

**DOI:** 10.1055/a-2724-3558

**Published:** 2025-11-28

**Authors:** Judith Stumm, Lucie Schröder, Meta Herrmann, Jennifer Marie Burchardi, Sandra Fahrenkrog

**Affiliations:** 1 Institut für Medizinische Soziologie und Rehabilitationswissenschaft, Charité Universitätsmedizin Berlin

**Keywords:** Ambulante Rehabilitation, Kinder und Jugendliche, Adipositas, Herausforderungen, Erfolge, Fachpersonal, Outpatient rehabilitation, Children and adolescents, Obesity, Achievements, Challenges, Professionals

## Abstract

**Ziel der Studie:**

Ziel der Studie ist es, Ziele, Erfolge und Herausforderungen eines ambulanten Rehabilitationsprogramms für Kinder und Jugendliche mit Adipositas aus der Perspektive von Fachpersonal aus Reha-Einrichtungen zu explorieren.

**Methodik:**

Im Rahmen eines mehrstufigen Forschungsmodells wurden in einer Prozessevaluation 19 semistrukturierte leitfadengestützte Telefoninterviews mit dem medizinischen und therapeutischen Fachpersonal aus vier Rehazentren geführt und inhaltsanalytisch mit der Framework Analyse analysiert.

**Ergebnisse:**

Die Interviewten sind sich einig darüber, dass das Ziel eine langfristige und nachhaltige Veränderung des Lebensstils ist. Der Gewichtsverlust spielt darüber hinaus sowohl für das Ziel als auch für den Erfolg des Rehaprogramms eine wichtige Rolle. Der langfristige Transfer des Gelernten in den Alltag, die wahrgenommene geringe Motivation der Eltern sowie die schulbegleitende Phase des Programms stellen eine Herausforderung dar. Die (aktive) Rolle der Eltern in dem ambulanten Rehaprogramm ist geprägt durch unterschiedliche Erwartungshaltungen aller Akteur*innen und wird beeinflusst durch (nicht) vorhandene Ressourcen der Familien. Die geringe Teilnehmer*innenzahl ist vermutlich auf den geringen Bekanntheitsgrad sowie die geringe Anzahl ambulanter Rehaprogramme bundesweit zurück zu führen.

**Schlussfolgerung:**

Das ambulante Rehaprogramm für Kinder und Jugendliche mit Adipositas weist individuelle Erfolgspotentiale auf. Die Ziele der Reha sollten jedoch transparenter kommuniziert werden, insbesondere in Bezug auf möglichen Gewichtsverlust, der wiederum Einfluss auf die Erwartungshaltung und Motivation der Kinder und Jugendlichen sowie ihrer Eltern hat. Durch einen spezifischeren Einbezug der Eltern können verschiedene Herausforderungen überwunden und das Gelernte effektiver in den Alltag transferiert werden.

## Abstract (en)

**Purpose:**

The aim of the study was to explore the goals, achievements and challenges of an outpatient rehabilitation program for obese children and adolescents from the perspective of specialist staff at rehabilitation centres.

**Methods:**

As part of a multi-stage research project, 19 semi-structured, guideline-based telephone interviews were conducted with the medical and therapeutic staff of four rehabilitation centres and analysed using Framework Analysis.

**Results:**

The interviewees agreed that the goal was a long-term and sustainable change in lifestyle. Furthermore, weight loss played an important role in both the goal and success of the rehabilitation program. Implementation of lessons learned into everyday life on a long-term basis, lower motivation of parents and various difficulties encountered by rehabilitants and staff when concomitant measures of the program were carried out during weeks of school term posed a challenge. The (active) role of parents in the outpatient rehabilitation program was shaped by different expectations on the part of all stakeholders and influenced by resources available to the families to different extents. The low number of participants was probably due to the low level of awareness and the small number of outpatient rehabilitation programs in Germany.

**Conclusion:**

The outpatient rehabilitation program for obese children and adolescents has potential for success in individual cases. However, the goals of the rehabilitation program should be communicated more transparently, especially with regard to possible weight reduction, which in turn influences the expectations and motivation of children and adolescents and their parents. By involving parents more specifically, various challenges can be addressed and the lessons learned can be implemented more effectively into everyday life.

## Einleitung

Daten aus der zweiten Erhebungswelle der KiGGS-Studie des Robert Koch-Instituts von 2014–2017 zeigen zwar eine sich stabilisierende, jedoch immer noch hohe Prävalenz von Übergewicht und Adipositas bei Kindern und Jugendlichen (KiJu). Aus den Daten geht hervor, dass in Deutschland insgesamt 15,4% der KiJu zwischen 3 und 17 Jahren an Übergewicht leiden; davon 4,78% an Adipositas und 1,12% an schwerer Adipositas 1 . Insbesondere durch die Covid-19-Pandemie – mit ihren durch den Lockdown einhergehenden Einschränkungen – wurden die Auswirkungen eines adipogenen Umfelds der Familien verstärkt 2 3 .

Übergewicht und vor allem Adipositas bei KiJu sind langfristig mit negativen Gesundheitsoutcomes assoziiert. Im Vergleich haben Kinder mit Adipositas deutlich häufiger erhöhten Blutdruck, Fettstoffwechselstörungen sowie Störungen des Glukosestoffwechsels 4 . Darüber hinaus erhöht ein hoher Body-Mass-Index (BMI) bei KiJu das Risiko, im Verlauf des Erwachsenenalters an Diabetes Typ 2, Bluthochdruck und Herz-Kreislauf-Erkrankungen zu erkranken 5 . Schließlich nehmen die beschriebenen Faktoren einen ungünstigen Einfluss sowohl auf das körperliche als auch auf das psychische Wohlbefinden der KiJu und führen somit zu einer deutlich verminderten Lebensqualität 6 .

Ein sektorenübergreifender Ansatz spielt in der Versorgung von Übergewicht und Adipositas eine wichtige Rolle. Neben den Kinder- und Jugendärzt*innen sowie den Hausärzt*innen der Familien 7 , ist die medizinische Rehabilitation für KiJu ein bedeutendes Versorgungselement 8 9 . Diese orientiert sich am biopsychosozialen Modell der Internationalen Klassifikation der Funktionsfähigkeit, Behinderung und Gesundheit (ICF).

Mit der Verabschiedung des Gesetzes zur Flexibilisierung des Übergangs vom Erwerbsleben in den Ruhestand und zur Stärkung von Prävention und Rehabilitation im Erwerbsleben (Flexirentengesetz) wurde die Möglichkeit eröffnet, Leistungen zur KiJu-Rehabilitation auch ambulant zu erbringen. Ambulante Rehabilitationsstrukturen sind wohnortnah und interdisziplinär. Sie unterscheiden sich von der stationären Rehabilitation beispielsweise durch die unmittelbare Einbeziehung von Kontextfaktoren, wie z. B. schulischer bzw. ausbildungsbezogener Aspekte 10 . Weitere Vorteile von ambulanten Therapiemaßnahmen sind die einfache Einbindung der Familie, geringere Kosten und die Behandlung im gewohnten Umfeld der Kinder und Jugendlichen. Dadurch können Alltagsschwierigkeiten und Rückfälle individuell besser berücksichtigt werden 7 . Das Angebot von ambulanten multidisziplinären Therapieprogrammen, insbesondere ambulanten Rehabilitationsmaßnahmen für KiJu, ist jedoch bundesweit nicht flächendeckend etabliert 2 .

Aktuell existieren bundesweit lediglich fünf ambulante Rehabilitationszentren, die durch die Deutsche Rentenversicherung zugelassen sind. Entsprechend liegen hierzu noch keine Wirksamkeitsstudien vor. Ältere Studien von 2003–2006 zu anderen ambulanten Therapieangeboten für Kinder und Jugendliche zeigen positive, auch langfristige Effekte in Hinblick auf Gewichtsreduktion, Reduktion des BMI, Gesundheitsverhalten (Bewegung, Ernährung und Essverhalten) und Lebensqualität 11 12 13 . Eine niedrigschwellige, multidisziplinäre ambulante Intervention über einen Zeitraum von 12 Monaten zeigte außerdem eine Verbesserung im Selbstwert und bei emotionalen Problemen der KiJu zum Ende der Maßnahme 12 .

Im Rahmen eines geförderten Projektes wird das ambulante Rehabilitationsprogramm für Kinder und Jugendliche mit Adipositas (KiJu) von vier Rehazentren in Deutschland hinsichtlich seiner Wirksamkeit, Optimierungsmöglichkeiten und Zufriedenheit der Teilnehmenden evaluiert.

Das Programm richtet sich an 6- bis 18-jährige KiJu mit der Diagnose Übergewicht und Adipositas. Ein Elternteil übernimmt jeweils die Rolle der/des Co-Rehabilitand*in während eines Behandlungszeitraums von 13 Wochen. An einer ersten Intensivwoche über fünf Tage à sechs Stunden nehmen sowohl KiJu als auch ihre Eltern teil und absolvieren gemeinsame Therapieeinheiten, u. a. zu den Themen Bewegung, Ernährung und Sozialkompetenz.

In der anschließenden zwölfwöchigen schulbegleitenden Phase kommen KiJu an zwei Nachmittagen pro Woche in das Rehazentrum und besuchen Gruppenkurse wie z. B. Kochen, Sport und psychologische Gruppengespräche und Psychoedukation. Die Elternteile nehmen ab der zweiten Woche parallel einmal wöchentlich an Bewegungs- und Ernährungstherapien sowie Sozialkompetenz-Schulungen teil. Sowohl die KiJu als auch die Eltern werden über den gesamten Zeitraum hinweg durch ein multidisziplinäres Team betreut.

In einem mehrstufigen Forschungsmodell wurden im Rahmen einer Prozessevaluation u. a. qualitative Interviews mit am Rehabilitationsprogramm beteiligtem Fachpersonal eingebettet, mit dem Ziel, ihre Perspektive zu den Zielen, Erfolgen und Herausforderungen des Programms zu explorieren. In diesem Artikel werden ausschließlich die Ergebnisse der beschriebenen qualitativen Erhebung bei dem Fachpersonal dargestellt.

## Material und Methoden

### Studiendesign

Die semistrukturierten Leitfadeninterviews mit dem Fachpersonal (n=19) aus vier Rehabilitationszentren waren Teil einer übergeordneten kontrollierten zweiarmigen Evaluationsstudie mit einem parallelen mixed-methods-Design. Die übergeordnete Studie bestand aus einer Ergebnisevaluation zur Wirksamkeit des Programms mit einer Fragebogenerhebung zu Beginn und zum Ende der Rehamaßnahme sowie 6 Monate nach Beendigung und einer Prozessevaluation, in welcher neben den Interviews mit dem Fachpersonal auch Interviews mit Eltern und Fokusgruppen mit teilnehmenden KiJu durchgeführt wurden. Die Ergebnis- und die Prozessevaluation wurden parallel zueinander durchgeführt. Insgesamt wurden 74 KiJu in die Interventionsgruppe eingeschlossen, wobei aufgrund von 4 Dropouts in einem Rehazentrum die geplante Stichprobengröße von 78 nicht erreicht wurde. Zur Beschreibung des methodischen Vorgehens wird die COREQ-Checkliste verwendet ( **Anhang 1** ) 14 .

Es wurde eine qualitative Vorgehensweise gewählt, um die subjektiven Perspektiven des Fachpersonals bezogen auf das Programm selbst, aber auch auf die Zielgruppe des Programms tiefergehend zu beleuchten 15 .

### Ein- und Ausschlusskriterien

Zwischen August und Dezember 2023 wurde unterschiedliches Fachpersonal der vier Rehazentren für die Interviews eingeschlossen, die medizinisch und/oder therapeutisch in das Rehabilitationsprogramm aktiv involviert waren sowie mindestens ein Jahr Berufserfahrung in der medizinischen KiJu-Rehabilitation mitbrachten. Ziel war es, die Perspektiven möglichst aller Berufsgruppen, die in dem Rehabilitationsprogramm tätig sind, einzubeziehen.

Bei allen vier Rehabilitationszentren handelt es sich um ambulante Rehazentren, die sowohl Kinder- als auch Erwachsenenrehabilitation durchführen. Nur eines der 4 Rehabilitationszentren bietet noch zusätzlich ein stationäres Rehabilitationsprogramm im Bereich der Orthopädie bei Erwachsenen an.

Alle vier kooperierenden Rehazentren wandten das in seiner Struktur und seinem Aufbau gleiche Programm an, welches in der spezifischen Durchführung durchaus, beispielswiese aufgrund von Standortfaktoren, Personalausstattung sowie finanziellen und materiellen Ressourcen, minimal voneinader abweichen konnte.

### Rekrutierung

Das interviewte Fachpersonal wurde bewusst durch die jeweiligen koordinierenden bzw. leitenden Mitarbeiter*innen der entsprechenden Rehazentren ausgewählt, um eine heterogene Teilnehmer*innen-Gruppe hinsichtlich des Alters, des Geschlechts, der Fachdisziplin und der Berufserfahrung zu rekrutieren. Die Terminvereinbarung wurde ebenso über die jeweiligen leitenden Mitarbeiter*innen koordiniert. Alle angefragten Mitarbeiter*innen der Rehazentren willigten ein, an einem Interview teilzunehmen.

Die Interviewteilnehmer*innen wurden vor Beginn des Interviews sowohl schriftlich als auch mündlich über die Studie informiert und gaben ihre schriftliche Einwilligung zur Erhebung der Daten in anonymisierter Form.

### Leitfadenerstellung, Datenerhebung und Stichprobe

Der Leitfaden ( **Anhang 2** ) wurde in einem iterativen Prozess im Studienteam entwickelt und im Verlauf im Dialog angepasst und offen angewendet. Hierzu diente die Vorgehensweise von Helfferlich et al. (2019) sowie die 4-Schritte-Formel „SPSS“ (Sammeln, Prüfen, Sortieren, Subsummieren) zur methodischen Orientierung 16 . Hauptthemen waren das Rehaprogramm, die Zusammenarbeit mit den KiJu und ihren Eltern, die Optimierung des Programms und die Gewichtsdiskriminierung. Der Leitfaden wurde in einem ersten Schritt mit medizinischem Fachpersonal aus dem eigenen Forschungsinstitut pilotiert und angepasst. Nach der Pilotierung des ersten Interviews mit Fachpersonal aus den Rehaeinrichtungen sowie im Verlauf wurden kleinere Änderungen zur inhaltlichen Präzision sowie an der Struktur des Leitfadens vorgenommen.

Die Interviews wurden telefonisch geführt und dauerten im Schnitt 50 Minuten (Range: 22–95). Sie wurden hauptsächlich durch eine Forscherin (studentische Hilfskraft/Medizinstudentin) im Rahmen einer Promotionsarbeit geführt. Eine Durchführung von Face-to-face Interviews konnte aufgrund der geografischen Entfernungen der 4 verschiedenen Rehaeinrichtungen und der zeitlich eingeschränkten Verfügbarkeit des Fachpersonals nicht umgesetzt werden.

Es wurden keine Interviews wiederholt geführt. Während des Prozesses der Interviewführung wurden Feldnotizen erstellt. Weiterhin fand ein regelmäßiger Austausch und eine Reflektion zwischen der interviewenden Forscherin und dem Studienteam (Postdoktorandin, Public Health; erfahrene Wissenschaftlerin, Psychologin und Public Health, Promovendin, Ethnologin) statt. Nach der Durchführung von 19 Interviews wurde schließlich von einer theoretischen Sättigung ausgegangen.

Die interviewende Forscherin stand zum Zeitpunkt der Interviewführung in keiner Beziehung zu den interviewten Personen. Diese wussten lediglich, dass es sich um Forscherinnen einer Forschungsinstitution handelte und ein großes Forschungsinteresse an dem Thema bestand. Der Ort, an dem sich die interviewten Personen zum Zeitpunkt aufhielten, wurde außerdem nicht erfragt. Wichtig war nur, dass sie ungestört waren.

### Datenauswertung

**Abb. 1:**
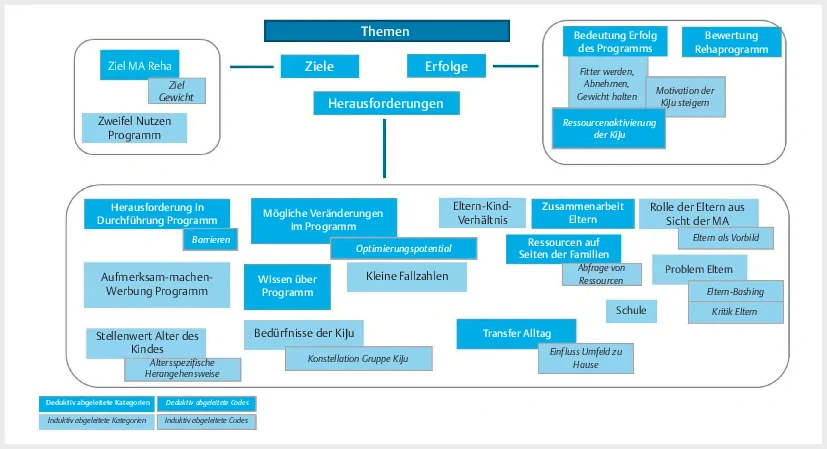
Darstellung ausgewählter Kategorien und Codes.

Die Interviews wurden audiodigital aufgenommen und in Orientierung an den vereinfachten Transkriptionsregeln nach Dresing und Pehl transkribiert 17 . Die Interviewten hatten anschließend keine Einsicht in die Transkripte ihres geführten Interviews, so dass diese kein Feedback zu den Ergebnissen geben konnten. Die Auswertung erfolgte inhaltsanalytisch mit der Frameworkanalyse 18 im Forscherinnenteam kombiniert deduktiv und induktiv ( Abb. 1 ) mit Unterstützung des Software-Programms Maxqda 2022 (VERBI Software GmbH, Deutschland). Die deduktiv abgeleiteten Kategorien und Codes wurden aus dem theoretischen Vorverständnis und den theoretischen Vorannahmen der Forscherinnen sowie der Interaktion im Forschungskontext abgeleitet. Die Frameworkanalyse unterliegt keinem theoretischen Ansatz. Sie ist vielmehr ein flexibles Instrument, welches auf die Generierung von Themen abzielt und auf verschiedene qualitative Ansätze angepasst werden kann.

Ein positives Votum für die Durchführung des Projektes wurde durch die Ethikkommission der Charité erteilt.

## Ergebnisse

Unter den interviewten Mitarbeiter*innen der ambulanten Rehazentren waren Sportwissenschaftler*innen, Orthopäd*innen, Psycholog*innen, Gesundheitsmanager*innen, Gymnastiklehrer*innen, Sporttherapeut*innen, Kinderkrankenpfleger*innen, Physiotherapeut*innen, Diätassistent*innen, Ernährungsberater*innen, Sozialarbeiter*innen und Kinderärzt*innen.

In Tab. 1 werden die soziodemografischen Daten des medizinisch-therapeutischen Fachpersonals aufgeführt.

**Table:** **Tab. 1** Darstellung soziodemografischer Daten von medizinischem und therapeutischen Fachpersonal der Rehazentren.

Soziodemografische Daten der Interviewteilnehmer*innen (n=19)	
Alter (Mittelwert; Median)	42 Jahre (SD: 11,9) ; 41 Jahre (Min: 23, Max: 60)
Geschlecht	weiblich: n=15
Berufsbezeichnungen	
Ärzt*in	*n=2*
Psychotherapeut*in	*n=1*
Psycholog*in	*n=3*
Gymnastiklehrer*in	*n=1*
Sporttherapeut*in	*n=2*
Gesundheitsmanager*in	*n=1*
*Pflegefachkraft*	*n=3*
Physiotherapeut*in	*n=1*
Diätassistent*in/ Ernährungsberater*in	*n=4*
Sozialarbeiter*in	*n=1*
durchschnittliche Beschäftigungsdauer in Reha-Einrichtung (in Jahren)	5 Jahre (Range: 1–15)
Aufteilung auf Rehazentren	Reha 1: n=4; Reha 2: n=7; Reha 3: n=5; Reha 4: n=3
**Kooperierende Rehazentren**	
Rehazentrum 1	Ambulant; Zielgruppe: Kinder und Erwachsene
Rehazentrum 2	Ambulant; Zielgruppe: Kinder und Erwachsene
Rehazentrum 3	Ambulant; Zielgruppe: Kinder und Erwachsene
Rehazentrum 4	ambulant, stationär; Zielgruppe: Kinder und Erwachsene

Die Ergebnisse werden mit ausgewählten Zitaten untermauert, welche für das bessere Verständnis und die bessere Lesbarkeit geglättet wurden. Weitere Zitate sind in **Anhang 3** zu finden.

Aus den Interviews mit dem Fachpersonal der vier Rehazentren werden folgende ausgewählte Themen der zugrundeliegenden Kategorien aus der Inhaltsanalyse dargestellt:

Ziele, Erfolge und Herausforderungen des ambulanten Rehaprogramms.

### Ziele des ambulanten Rehaprogramms

In der Beschreibung der Ziele lässt sich zunächst eine einheitliche rote Linie bei dem interviewten Fachpersonal ableiten. Demnach ist die Gewichtsreduktion nicht das primäre Ziel.

Vielmehr spielt die langfristige und nachhaltige Lebensstilveränderung der KiJu und ihrer Familien eine entscheidende Rolle. Der zu verändernde Lebensstil impliziert eine gesündere Lebensweise bestehend aus einer ausgewogenen Ernährung und viel Bewegung im Alltag. Voraussetzung für eine Veränderung im Alltag ist es, Verständnis für den (Umgang mit dem) eigenen Körper zu bekommen.

*„Ja, also das Ziel ist auf jeden Fall eine verbesserte Lebensweise und eine gesündere Lebensweise der Kinder und auch der Familien, also weil mit einfach ausgewogener Ernährung und viel Bewegung mit Alltag.“*

Trotzdem ist die Dimension Gewicht allgegenwärtig und wird, entgegen dem eigentlichen Rehaziel, zwischen den Zeilen als messbarer Indikator für den individuellen sowie von außen wahrzunehmenden Erfolg immer wieder thematisiert.

*„Naja (…) also das Gewicht ist uns erstmal relativ. Wir sind froh, wenn sie [KiJu] das Gewicht HALTEN über die Zeit wo sie hier sind, weil die meisten doch immer ein bisschen wachsen oder an Muskelmasse zulegen, dafür aber an Fett verlieren. Also das Gewicht, klar, ist irgendwo ein Indikator. Der steht irgendwo auf dem Papier, der wird irgendwo als Relevanz benutzt aber im Endeffekt ist ja irgendwo auch das Äußerliche, dieses Ansehen, ne.“*

### Erfolge des ambulanten Rehaprogramms

Der Erfolg wird durch das Fachpersonal anhand von vielen unterschiedlichen individuellen Faktoren gemessen. Die Mitgliedschaft in einem (Sport-)Verein beispielsweise trägt aus Sicht des Fachpersonals nicht nur zu mehr Bewegung bei, sondern auch dazu, dass die KiJu eine Beschäftigung haben, weniger einsam sind und sich weniger in der digitalen Medienwelt isolieren. Die Abschlussuntersuchung sowie das Abschlussgespräch im Rehazentrum sind u. a. ausschlaggebend für die Messung des Erfolgs. Neben der zahlenmäßigen Erfassung des BMI ist der Motoriktest darüber hinaus ein Maß für Erfolg. Für das Fachpersonal zeigt sich ein Erfolg des Programms auch in einer sichtbaren Verbesserung im Kraft- und Ausdauerbereich, der zu sportlichen Erfolgen im Alltag führt. Auch die positiven Gespräche mit den Familien und die daraus hervorgehende Motivation, das Gelernte im Alltag umzusetzen, stellt einen Erfolgsfaktor für das Fachpersonal dar. Offensichtlicher Spaß an Bewegung und ein positiver Bezug zum Essen wird ebenso als Erfolg des Programmes gewertet.

*„(…), dass sie einfach verstehen, wo sie was verändern können für die Zukunft. Wenn sie erzählen, dass sie ja, kleine Veränderungen vorgenommen haben, dass sie vielleicht einen bewegteren Alltag haben, vielleicht auch mehr mal selber was kochen, was ausprobieren. Wenn sie eine Brotdose mit zur Schule nehmen, da sehe ich schon diese kleinen Erfolge, das sehe ich da einfach eher in den Vordergrund gerückt. Und langfristige Veränderung einfach.“*

### Herausforderungen des ambulanten Rehaprogramms

#### Schulbegleitende Phase

Beinahe alle Interviewten thematisierten die schulbegleitende Phase als Herausforderung. Für die KiJu ist aus Sicht des Fachpersonals die Reha am Nachmittag nach dem Schulbesuch besonders herausfordernd, da sie erkennbar müde und erschöpft sind. Das Fachpersonal äußert die Wahrnehmung, dass es den KiJu in der schulbegleitenden Phase schwerfällt, sich auf die Inhalte des Rehaprogramms zu konzentrieren. Es wird berichtet, dass die KiJu in einigen Fällen nach der Reha zusätzlich Hausaufgaben für den nächsten Schultag erledigen müssten.

*„Die Motivation der Kinder. Am Anfang sind die immer ganz begeistert und dann wird es irgendwann anstrengend. Es ist ja auch wirklich viel, da immer Intensivwoche und dann ist ja wochenlang immer nach der Schule. Das heißt, die müssen ja trotzdem zur Schule gehen, die müssen lernen, die müssen Hausaufgaben machen, Klausur schreiben und dann müssen sie quasi zu uns, um wieder Sport zu machen. Ja also die Motivation, die lässt irgendwann so ein bisschen nach.“*

#### Zusammensetzung der KiJu-Gruppen

Die Zusammensetzung der Gruppen stellt ebenso eine Herausforderung dar. Unterschiedliche Altersgruppen oder verschiedene kognitive und gesundheitliche Voraussetzungen und Bedürfnisse erschweren und überfordern laut der Interviews mit dem Fachpersonal die Gestaltung und Durchführung des Programms.

*„Eine Herausforderung ist tatsächlich, dass jede Gruppe von der Zusammenstellung anders ist. Manche teilweise mit Lernförderbedarf, dann gibt es Kinder mit Allergien, da müssen wir Rücksicht nehmen, dass wir da natürlich gerade in der Lehrküche die Zutaten, die Unverträglichkeiten hervorrufen, weglassen. Dann auch die Belastung, die das für die Familien bedeutet, das ist für die hierher zu kommen ja auch ein Stressfaktor. Den aufzufangen und versuchen, dann umzuleiten. Aber ich glaube tatsächlich die größte Schwierigkeit ist das, auf die einzelnen Bedürfnisse einzugehen.“*

#### Einfluss der Eltern im ambulanten Rehaprogramm

Die Bedeutung der Eltern wurde wiederholt durch das Fachpersonal adressiert. In beinahe allen Themen und Kategorien spielten die Familien bzw. Eltern eine entscheidende Rolle.

Aus Sicht des interviewten Fachpersonals scheint die Zusammenarbeit mit den Eltern unausweichlich für die Erreichung der Rehaziele. Der familiäre und soziale Kontext sei besonders relevant und hat Einfluss auf die nachhaltige Verhaltensänderung der KiJu. Demnach würde die Aufgabe der Eltern vielmehr beinhalten, als nur ihr Kind zur Reha zu „schicken“. Das Fachpersonal beschreibt, dass die Eltern in ihrer Rolle als Vorbild durch ihre Motivation, Teilnahme an dem Programm und langfristigen Veränderungen im häuslichen Umfeld großen Einfluss auf die Motivation und das Verhalten ihrer Kinder nehmen.

Die individuellen Lebenswelten, Ressourcen, möglichen gesundheitlichen Beeinträchtigungen und anderen Belastungen der Familien beeinflussen darüber hinaus die Zusammenarbeit mit Eltern und sind durch das Programm häufig schwer zu kompensieren. Insbesondere für diese individuellen Faktoren bringt das Fachpersonal nur wenig Verständnis auf und kritisiert diese. Durch das Fachpersonal geäußerte Erwartungen an die Eltern scheinen aus ihrer Sicht nicht erfüllt zu werden.

*„Ich finde es total wichtig, dass wir mit den Eltern zusammenarbeiten. Also rein mit den Kindern und Jugendlichen, das kann nur ein Teilerfolg geben. Aber die Eltern müssen wir mit ins Boot holen, deshalb finde ich es total wichtig mit ihnen zusammenzuarbeiten. Aber sie (Eltern) sind sehr unterschiedlich, haben ganz unterschiedliche soziale Hintergründe, haben auch zum Teil Erkrankungen, in die wir gar nicht so, was wir nur so nebenher mitbekommen oder auch familiäre Belastungen, was wir hier gar nicht auffangen können.“*

#### Mangelnder Bekanntheitsgrad des ambulanten Rehaprogramms

Alle vier Rehazentren hatten bislang trotz eigeninitiierter Werbung, Vernetzung mit anderen Einrichtungen im Gesundheitssektor und Informationsveranstaltungen eher geringe Teilnehmer*innenzahlen. Sowohl das Wissen über das Bestehen eines ambulanten Rehaprogramms als auch der Mehrwert des Programms scheinen laut den interviewten Mitarbeiter*innen gleichwohl zahlreicher Bemühungen um mehr Bekanntheit bei vielen potentiell involvierten Akteur*innen noch nicht durchgedrungen zu sein.

*„Wir haben einen Fahrdienst, holen die Zuhause ab etc. nehmen auf die Schule Rücksicht. Ich glaube es ist eher das Wissen, dass es überhaupt Reha-Programme gibt für Jugendliche und, dass es auch ambulante Reha-Möglichkeiten gibt, ich glaube da ist das Hauptproblem, dass es einfach nicht bekannt ist.“*

#### Transfer in den Alltag

Der Transfer in den Alltag bereitet dem Fachpersonal der Rehazentren Sorge. Über die Umsetzung des Gelernten in den Alltag sowie die Nachhaltigkeit des Programms bzw. der Verhaltensänderung bleibt das Fachpersonal meist im Ungewissen, wenn die KiJu nicht im Anschluss an einem Nachsorgeprogramm im selben Rehazentrum teilnehmen. Laut den Interviewten lässt die anfängliche Motivation der KiJu und ihrer Familien im Verlauf des Rehaprogramms nach und die KiJu und ihre Familien fallen erneut in alte Muster.

Eine Möglichkeit, die nachhaltige Verhaltensänderung sicherzustellen, wäre laut dem Fachpersonal eine Verlängerung des Programms oder auch fortlaufende gemeinsame Aktivitäten im Rehazentrum, wie gemeinsames Sporttreiben oder Kochen.

*„Es ist die Nachhaltigkeit, die mir immer noch Sorgen bereitet. Bleiben die Erfolge, die wir während der Reha erzielen? Und wir haben es auch bisher tatsächlich nicht geschafft nochmal eine Familie dazu zu bewegen das Ganze nochmal zu machen. Und das ist was, was im Moment für mich noch eine Baustelle ist.“*

## Diskussion

Im Rahmen einer Interviewstudie mit dem Fachpersonal aus vier Rehabilitationszentren, welche ein von Struktur, Aufbau und Inhalt gleiches ambulantes Rehaprogramm für KiJu mit Adipositas umsetzen, wurden Perspektiven zu Zielen, Erfolg und Herausforderungen des ambulanten Rehaprogramms näher beleuchtet.

### Ziele des ambulanten Rehaprogramms

Aus den Interviewergebnissen geht hervor, dass eine Veränderung des Lebensstils als primär kommuniziertes Ziel des ambulanten Rehaprogramms häufig nicht den Erwartungen der Gewichtsreduktion der KiJu und ihren Familien entspricht. Aus einem systematischen Review von Mühlig et al. 19 zu Gewichtsreduktion in konservativen Therapieansätzen für KiJu mit Adipositas geht als Ergebnis nur eine geringe Gewichtsreduktion hervor. Hohe Dropout-Raten, insbesondere in den Follow-up-Erhebungen untersuchter Interventionen ergeben sich u. a. daraus, dass KiJu und ihre Familien mit hohen Erwartungen in Bezug auf eine Gewichtsreduktion die Therapie beginnen und diese nicht erfüllt werden. Weiterhin besteht ebenso die Gefahr, dass die KiJu die ausbleibende Gewichtsreduktion als persönliches Versagen interpretieren 19 . Folgen können ein vermindertes Selbstbewusstsein, gestörtes Essverhalten bis hin zu Depressionen sein 20 . Aus diesem Grund ist zu empfehlen, die Familien und KiJu, die an einem Adipositasprogramm teilnehmen, vorab explizit und umfassend über die Ziele des Programms und den relativ geringen bis ausbleibenden Gewichtsverlust informiert werden 19 . Auch das Fachpersonal sollte die kommunizierten Ziele klar und transparent verfolgen, so dass diese nicht durch KiJu und ihre Eltern missverstanden werden können.

Insgesamt sollte das primär kommunizierte Ziel der Veränderung des Lebensstils insbesondere in der ambulanten Rehabilitation spezifischer verfolgt werden, da dort Barrieren im Alltagstransfer zeitnah festgestellt und bewältigt werden können. Die Lebensstilanpassung kann simultan umgesetzt und nachhaltig unterstützt werden.

### Erfolge des ambulanten Rehaprogramms

Der Faktor Spaß während der Teilnahme an dem Programm und der Umsetzung des Gelernten in den Alltag wird durch das interviewte Fachpersonal als (Teil-)Erfolg des Programms gewertet. Das durchgeführte RCT von Aguilar-Cordero et al. 21 zeigt, dass sich neben der Reduzierung des Fettanteils in der Studiengruppe, welche sowohl physische Aktivitäten als auch Ernährungsberatung beinhaltete, ebenso die Lebensqualität (gemessen mit dem HRQoL) signifikant im Gegensatz zur Kontrollgruppe (keine physische Aktivität) verbessert hatte 21 . Die gesundheitsbezogene Lebensqualität sollte in dem Rehaprogramm für KiJu mit Adipositas unbedingt ein Maß für den angestrebten Erfolg des Programms darstellen. Insbesondere KiJu mit Adipositas weisen laut Baumgarten et al. 6 im Vergleich zu KiJu mit anderen chronischen Erkrankungen Einbußen in der gesundheitsbezogenen Lebensqualität auf 6 .

### Herausforderungen des ambulanten Rehaprogramm

#### Schulbegleitende Phase

Eine Besonderheit der ambulanten Rehabilitation ist die Möglichkeit, wohnortnah und an den individuellen Kontext angepasste Leistungen flexibel zu erbringen. Hierzu zählt die Einbindung schulbezogener Faktoren sowie die aktive Beteiligung von Bezugspersonen 22 . Aus den Interviews wird jedoch die schulbegleitende Phase im Programm als Herausforderung benannt, die mit einer erhöhten Belastung der KiJu einhergeht. Diese Herausforderungen scheinen das Programm noch zu überfordern.

Diese erhöhte Belastung in der schulbegleitenden Phase sollte zukünftig verringert werden. So könnten strukturelle Anpassungen des Programms hinsichtlich der besseren Durchführbarkeit, wie beispielsweise die Anpassung der Rehazeiten, die Freistellung von Hausaufgaben oder die Verlagerung eines Rehatags auf das Wochenende, diese Barrieren langfristig überwinden.

#### Einfluss der Eltern im ambulanten Rehaprogramm

Induktiv lässt sich aus den Interviews eine Kritik an den Eltern bzw. den Familien der KiJu ableiten. Diese Kritik wird in Hinblick auf eine teilweise geringe Motivation und Eigenverantwortung der Eltern in dem gesamten Versorgungsprozess geäußert.

Das (soziale) Umfeld ist eine Hauptkomponente hinsichtlich des Ursprungs und Bestehens bestimmter Verhaltensmuster sowie einer möglichen zukünftigen Verhaltensänderung 23 . Die Berücksichtigung von individuellen Settings und Ressourcen sollte demnach unbedingt in die Gestaltung von ambulanten Rehabilitationsprogrammen Einzug finden. Die spezifische Abfrage von vorhandenen Ressourcen, die für eine nachhaltige Übertragung des Gelernten in den Alltag notwendig sind, sollte bestenfalls zu Beginn der Durchführung des Rehaprogramms stattfinden. Laut Empfehlungen der S3-Leitlinie Adipositas im Kindesalter sollten interdisziplinäre verhaltenstherapeutische Interventionen bei Kindern zusätzlich (separat) bei Eltern bzw. in der Familie durchgeführt werden; bei Jugendlichen sollten die Eltern aktiv miteinbezogen werden 7 .

In diesem Kontext ist wichtig zu beachten, dass KiJu aus Familien mit einem niedrigen sozioökonomischen Status häufiger von Adipositas betroffen sind als KiJu aus Familien mit einem hohen sozioökonomischen Status 1 24 . Insbesondere für Familien mit einem niedrigeren sozioökonomischen Status stellt das Format der ambulanten Reha durch seine Niedrigschwelligkeit ein geeigneteres Angebot im Vergleich zur stationären Rehabilitation dar.

#### Mangelnder Bekanntheitsgrad des ambulanten Rehaprogramms

Der geringe Bekanntheitsgrad sowohl unter medizinischem Personal als auch unter KiJu und ihren Familien selbst schlägt sich auf die Teilnehmer*innenzahlen nieder. Die insgesamt geringe Anzahl an ambulanten Rehazentren deutschlandweit, die KiJu-Rehabilitation anbieten 25 , hat vermutlich sowohl Einfluss auf den Bekanntheitsgrad solcher Programme an sich als auch auf den Bekanntheitsgrad der Rehazentren, die solche Programme durchführen. Die Stärken einer ambulanten Reha sollten in diesem Zusammenhang entsprechenden Zielgruppen und Akteur*innen näher gebracht werden. In diesem Zusammenhang sollten insbesondere die Vorteile für Familien, die aus zeitlichen Gründen das Kind nicht auf einen mehrwöchigen stationären Rehaaufenthalt begleiten können, hervorgehoben werden 25 .

#### Transfer in den Alltag

Das interviewte Fachpersonal thematisiert die Sorge um die Nachhaltigkeit der Therapieerfolge, sobald das Rehaprogramm endet und die KiJu an keinem Rehanachsorgeprogramm teilnehmen. Um nachhaltige gesundheitliche Effekte zu erzielen, braucht es vor allem hinsichtlich des Transfers in den Alltag Umfeld- und gemeindenahe Ansätze 26 .

Die Herausforderung der nachlassenden Motivation der Teilnehmenden und ihrer Familien im Verlauf der ambulanten Reha, die die Nachhaltigkeit und den Transfer in den Alltag gefährdet, gilt es im Rahmen der Gestaltung des Therapieprogramms kritisch zu hinterfragen und adäquate Implikationen hierfür zu entwickeln. Vorstellbar wäre hier ebenso eine intensivierte und individualisierte Einbindung der Eltern und Familien. Die Motivierende Gesprächsführung könnte hierbei ebenso bereits während des Programms eine geeignete Methode darstellen, um der nachlassenden Motivation der KiJu und ihrer Eltern vorzubeugen 27 .

### Limitationen

Das interviewte Fachpersonal wurde durch die jeweilige Rehaeinrichtung selbst ausgesucht, so dass hier zu vermuten ist, dass insbesondere die engagierten und interessierten Mitarbeiter*innen angesprochen wurden. Möglicherweise konnte dadurch kein vollumfänglich heterogenes Meinungsspektrum abgebildet werden. Wenngleich eine heterogene Auswahl der Interviewteilnehmer*innen beabsichtigt war und gezielt durch die Studienkoordination der jeweiligen Einrichtung vorgenommen wurde, kann aufgrund der Anonymisierung nicht abgebildet werden, dass sich die interviewten Professionen nicht gleich über die Rehazentren verteilen.

Des Weiteren ist kritisch zu betrachten, dass keine Daten dazu erhoben wurden, ob das interviewte Fachpersonal bereits in einer stationären Rehabilitationseinrichtung gearbeitet hat. So ließen sich möglicherweise die abgebildeten Zitate für die Leser*innen besser einordnen.

Die Rehaprogramme der unterschiedlichen Einrichtungen sind in ihrer Struktur, Aufbau und Inhalt gleich, unterscheiden sich jedoch in Einzelaspekten. Diese Unterschiede wurden in der Analyse der Interviews nicht spezifisch berücksichtigt, da dies für die Prozessevaluation nicht von Bedeutung ist.

In diesem Artikel wird nur eine von drei erhobenen Perspektiven berücksichtigt. Die Interpretation dieser Ergebnisse sollte daher ohne die Miteinbeziehung anderer Blickwinkel (der KiJu und ihrer Eltern) nur vorsichtig und unter Vorbehalt vorgenommen werden.

Die Durchführung von lediglich Telefon-Interviews schränkt möglicherweise die Tiefe des Gesagten durch die Interviewenden ein, da hierdurch die direkte Interaktion zwischen Interviewenden und Interviewten gestört wird und vermutlich ebenso die Flexibilität und die Vertrauenswürdigkeit der Interviewenden durch das Fehlen von non-verbaler Kommunikation begrenzt ist .

## Kernaussagen

Die Ergebnisse deuten darauf hin, dass das ambulante Rehaprogramm aus Sicht des Fachpersonals positive Effekte ausübt. So scheinen der Spaß an der Bewegung und die Motivation zu gesundem Ess- und Ernährungsverhalten gesteigert. Zudem sollten erreichbare Ziele wie Lebensstilveränderungen formuliert, aber auch die Möglichkeit ausbleibender Gewichtsreduktion transparent kommuniziert werden. Hierdurch könnte einem Motivationsverlust vorgebeugt werden. Es wird nahegelegt, die Eltern intensiver miteinzubeziehen. Dadurch könnten Barrieren, wie beispielsweise Motivationseinbrüche und mangelnde Zeitressourcen, besser bewältigt und das Gelernte effektiver und nachhaltig in den Alltag übertragen werden.

Schließlich wird empfohlen, Informationen über ambulante Rehaprogramme für KiJu mit Adipositas in der Versorgungslandschaft und darüber hinaus weitreichend gestreut werden, damit der Bekanntheitsgrad gesteigert wird und mehr KiJu dieses Angebot wahrnehmen.

## Zusätzliches Material

Zusätzliches Material Zusätzliches Material

## References

[1] Schienkiewitz A Damerow S Schaffrath Rosario A Body-Mass-Index von Kindern und Jugendlichen: Prävalenzen und Verteilung unter Berücksichtigung von Untergewicht und extremer Adipositas: Ergebnisse aus KiGG Welle 2 und Trends Bundesgesundheitsbl Gesundheitsforsch Gesundheitsschutz 2019 62 10.1007/s00103-019-03015-8 31529189

[2] Böttcher M Sergeyev E Stoltze A Überblick über die ambulanten interdisziplinären Schulungsprogramme für Kinder und Jugendliche mit Adipositas in Deutschland Kinder Jugendmed 2023 23 54 58 10.1055/a-1970-6916

[3] Browne N T Snethen J A Greenberg C S When pandemics collide: the impact of COVID-19 on childhood obesity J Pediatr Nurs 2021 56 90 98 10.1016/j.pedn.2020.11.004 33293199 PMC7657263

[4] Friedemann C Heneghan C Mahtani K Cardiovascular disease risk in healthy children and its association with body mass index: systematic review and meta-analysis BMJ 2012 345 e4759 10.1136/bmj.e4759 23015032 PMC3458230

[5] Llewellyn A Simmonds M Owen C G Childhood obesity as a predictor of morbidity in adulthood: a systematic review and meta-analysis Obes Rev Off J Int Assoc Study Obes 2016 17 56 67 10.1111/obr.12316 26440472

[6] Baumgarten F Cohrdes C Schienkiewitz A Gesundheitsbezogene Lebensqualität und Zusammenhänge mit chronischen Erkrankungen und psychischen Auffälligkeiten bei Kindern und Jugendlichen: Ergebnisse aus KiGGS Welle 2 Bundesgesundheitsbl Gesundheitsforsch Gesundheitsschutz 2019 62 1205 1214 10.1007/s00103-019-03006-9 31529184

[7] AWMF online Evidenzbasierte (S3-) Leitlinie der Arbeitsgemeinschaft Adipositas im Kindes- und Jugendalter (AGA) der Deutschen Adipositas-Gesellschaft (DAG) und der Deutschen gesellschaft für Kinder- und Jugendmedizin (DGKJ)Therapie und Prävention der Adipositas im Kindes und Jugendalter(August 2019). AWMF-Nr. 050-002. Online:https://register.awmf.org/de/leitlinien/detail/050-002Zugriff: 10.09.2024

[8] Deutsche Rentenversicherung Bund. Reha für Kinder und Jugendliche (2023). Online:https://www.deutsche-rentenversicherung.de/DRV/DE/Reha/Medizinische-Reha/Reha-fuer-Kinder-und-Jugendliche/reha-fuer-kinder-und-jugendliche_node.htmlZugriff: 14.01.2025

[9] Widera T Baumgarten E Druckenmüller A Kinder- und Jugendlichen-Rehabilitation – Stand und Perspektiven aus Sicht der Deutschen Rentenversicherung Rehabilitation 2017 56 91 102 10.1055/s-0043-103065 28395372

[10] Deutsche Rentenversicherung Bund. Eckpunkte für die ambulanten Leistungen zur Kinder- und Jugendlichenrehabilitation (2022). Online:https://www.deutsche-rentenversicherung.de/SharedDocs/Downloads/DE/Experten/infos_fuer_reha_anbieter/eckpunkte_ambulante_kinderreha_pdf.htmlZugriff: 10.09.2024

[11] van Egmond-Frohlich A Brauer W Goldschmidt H Effekte eines strukturierten ambulanten Weiterbehandlungsprogrammes nach stationärer medizinischer Rehabilitation bei Kindern und Jugendlichen mit Adipositas – Multizentrische, randomisierte, kontrollierte Studie Rehabilitation 2006 45 40 51 16468112 10.1055/s-2005-915368

[12] Barnow S Bernheim D Schröder C Adipositas im Kindes- und Jugendalter – Erste Ergebnisse einer multimodalen Interventionsstudie in Mecklenburg-Vorpommern Psychother Psych Med 2003 53 7 14 10.1055/s-2003-36480 12514762

[13] Reinehr T Kersting M Wollenhaupt A Evaluation der Schulung „OBELDICKS“ für adipose Kinder und Jugendliche Klin Padiatr 2005 217 1 8 15640963 10.1055/s-2004-816246

[14] Tong A Sainsbury P Craig J Consolidated criteria for reporting qualitative research (COREQ): a 32-item checklist for interviews and focus groups Int J Health Care 2007 19 9 10.1093/intqhc/mzm042 17872937

[15] Flick U Forschungsdesign. Prozess und Theorien in qualitativer ForschungIn:Flick U, Hrsg. Qualitative Sozialforschung – Eine Einführung. 5. Aufl Hamburg Rowohlt Taschenbuch Verlag 2012

[16] Helfferich C Leitfaden- und ExperteninterviewsIn:Baur N, Blasius J, Hrsg. Handbuch Methoden der empirischen Sozialforschung Wiesbaden Springer Nature 2019 669 686 10.1007/978-3-658-21308-4

[17] Dresing T Pehl T Praxisbuch Interview, Transkription & Analyse: Anleitungen und Regelsysteme für qualitativ Forschende Marburg Dresing 2015

[18] Gale N K Heath G Cameron E Using the framework method for the analysis of qualitative data in multi-disciplinary health research BMC Med Res Methodol 2013 8 10.1186/1471-2288-13-117 PMC3848812 24047204

[19] Mühlig Y Wabitsch M Moss A Weight loss in children and adolescents Dtsch Arzteblatt Int 2014 111 818 824 10.3238/arztebl.2014.0818 PMC4269075 25512008

[20] Crow S Eisenberg M E Story M Psychosocial and behavioral correlates of dieting among overweight and non-overweight adolescents J Adolesc Health 2006 38 569 574 10.1016/j.jadohealth.2005.05.019 16635769

[21] Aguilar-Cordero M J León Ríos X A Rojas-Carvajal A M Effects of physical activity on quality of life in overweight and obese children Nutr Hosp 2021 38 736 741 10.20960/nh.03373 34092077

[22] Deutsche Rentenversicherung Bund. Eckpunkte für die ambulanten Leistungen zur Kinder- und Jugendlichenrehabilitation. 2023https://www.deutsche-rentenversicherung.de/SharedDocs/Downloads/DE/Experten/infos_fuer_reha_anbieter/eckpunkte_ambulante_kinderreha_pdf.htmlZugriff: 01.10.2024

[23] Fisberg M Maximino P Kain J Obesogenic environment – intervention opportunities J Pediatr (Rio J) 2016 92 30 39 10.1016/j.jped.2016.02.007 27005593

[24] Bantel S Buitkamp M Wünsch A Kindergesundheit in der COVID-19-Pandemie: Ergebnisse aus den Schuleingangsuntersuchungen und einer Elternbefragung in der Region Hannover Bundesgesundheitsbl Gesundheitsforsch Gesundheitsschutz 2021 64 1541 1550 10.1007/s00103-021-03446-2 PMC8553103 34713308

[25] Stark C Jackels M Alberg E Pionierbereich: Ambulante Kinder- und Jugendrehabilitation Pflege Z 2020 73 30 33 10.1007/s41906-020-0764-1

[26] Weihrauch-Blüher S Kromeyer-Hauschild K Graf C Current guidelines for obesity prevention in childhood and adolescence Obes Facts 2018 11 263 276 10.1159/000486512 29969778 PMC6103347

[27] Miller W R Rollnick S Motivierende Gesprächsführung: Motivational interviewing. 3. Aufl Freiburg in Breisgau Lambertus-Verlag 2015

