# Supplementary material for: Potential and challenges of an outpatient rehabilitation programme for children and adolescents with obesity – Interviews with professionals from rehabilitation centres
Source: Rehabilitation (Stuttg). 2025 Nov 28;65(4):234–42. [Article in German] doi: 10.1055/a-2724-3558 (PMC13493175; doi:10.1055/a-2724-3558)
Supplement: Supplementary file 1 [file 10-1055-a-2724-3558-2025-01-0003-oa.pdf]

Zusätzliches Material

Anhang 1: COREQ (COnsolidated criteria for REporting Qualitative research) Checklist

Eine Checkliste mit Punkten, die in Berichten über qualitative Forschung enthalten sein sollten. Sie müssen die Seitenzahl in Ihrem Manuskript angeben, auf der Sie jeden der in dieser Checkliste aufgeführten Punkte behandeln. Wenn Sie diese Informationen nicht angegeben haben, überarbeiten Sie Ihr Manuskript vor der Einreichung entsprechend oder vermerken Sie „N/A".

Thema	Artikel Nr.	Leitfragen/Beschreibung	Vermerkt auf Seite
**Bereich 1: Forschungsteam und Reflexivität**			
Persönliche Eigenschaften			
Interviewer*in/Moderator*in	1	Welche/r Autor*in/Autor*innen hat/haben das Interview oder die Fokusgruppe durchgeführt?	4
Referenzen	2	Was waren die Qualifikationen des/der Forscher*in? Z. B. PhD, MD	4
Beschäftigung/Beruf	3	Was war die Art der Beschäftigung/Beruf zum Zeitpunkt der Studie?	4
Geschlecht	4	War der/die Forscher*in männlich oder weiblich?	4
Erfahrung und Ausbildung	5	Welche Erfahrungen oder Ausbildung hatte der/die Forscher*in?	4
*Beziehung zu den Teilnehmer*innen*			
Herstellung der Beziehung	6	Wurde vor Beginn der Studie eine Beziehung aufgebaut?	4
Kenntnisse der Teilnehmer*innen über den/die Interviewer*in	7	Was wussten die Teilnehmer*innen über den/die Forscher*in? Z. B. persönliche Ziele, Gründe für die Durchführung der Forschung	4
Merkmale des/der Interviewer*in	8	Welche Eigenschaften wurden über den/die Interviewer*in/Moderator*in berichtet? z. B. Voreingenommenheit, Annahmen, Gründe und Interessen in Bezug auf das Forschungsthema	4
**Bereich 2: Studiendesign**			
Theoretischer Rahmen			
Methodische Ausrichtung und Theorie	9	Welche methodische Ausrichtung wurde als Grundlage der Studie angegeben? Z. B. Grounded Theory, Diskursanalyse, Ethnografie, Phänomenologie, Inhaltsanalyse	4
*Teilnehmer*innenauswahl*			
Stichprobenauswahl	10	Wie wurden die Teilnehmer*innen ausgewählt? Z. B. gezielt, nach Zweckmäßigkeit, konsekutiv, Schneeballverfahren	3
Methodisches Vorgehen bei der Ansprache der Teilnehmer*innen	11	Wie wurden die Teilnehmer*innen angesprochen? Z. B. persönlich, per Telefon, Post, E-Mail	4
Stichprobengröße	12	Wie viele Teilnehmer*innen nahmen an der Studie teil?	2
Nicht-Teilnahme	13	Wie viele Personen haben die Teilnahme abgelehnt oder sind ausgestiegen? Gründe?	3
Setting			
Setting der Datenerfassung	14	Wo wurden die Daten erhoben? Z. B. zu Hause, in der Klinik, am Arbeitsplatz	4
Anwesenheit von Nicht-Teilnehmer*innen	15	Waren außer den Teilnehmer*innen und Forscher*innen noch andere Personen anwesend?	4
Beschreibung der Stichprobe	16	Welche sind die wichtigen Merkmale der Stichprobe? z. B. demografische Daten, Datum	5
Datenerhebung			
Interviewleitfaden	17	Wurden Fragen, Hinweise und Anleitungen von den Autor*innen bereitgestellt? Wurden diese vorab getestet?	3,4

Thema	Artikel Nr.	Leitfragen/Beschreibung	Vermerkt auf Seite
Wiederholte Interviews	18	Wurden wiederholte Befragungen durchgeführt? Wenn ja, wie viele?	4
Audio- und Videoaufnahmen	19	Wurden für die Datenerhebung im Rahmen der Studie Audio- oder Videoaufnahmen verwendet?	4
Feldnotizen	20	Wurden während und/oder nach dem Interview oder der Fokusgruppe Feldnotizen gemacht?	4
Dauer	21	Wie lange dauerten die Interviews oder Fokusgruppen?	4
Datensättigung	22	Wurde die Datensättigung diskutiert?	4
Vorlage Transkripte an Teilnehmer*innen	23	Wurden die Transkripte den Teilnehmer*innen zur Kommentierung und/oder Korrektur vorgelegt?	4
**Bereich 3: Analyse und Ergebnisse**			
Datenanalyse			
Anzahl der Kodierer*innen	24	Wie viele Kodierer*innen haben die Daten kodiert?	4
Beschreibung des Kodierbaums	25	Haben die Autor*innen eine Beschreibung des Kodierungsbaums bereitgestellt?	Abb.1
Ableitung der Themen	26	Wurden die Themen im Voraus festgelegt oder aus den Daten abgeleitet?	4
Software	27	Welche Software wurde gegebenenfalls zur Verwaltung der Daten verwendet?	4
Prüfung durch die Teilnehmer*innen	28	Haben die Teilnehmer*innen Feedback zu den Ergebnissen gegeben?	4
Ergebnisdarstellung			
Angeführte Zitate	29	Wurden Zitate der Teilnehmer*innen zur Veranschaulichung der Themen/Ergebnisse präsentiert? Wurde jedes Zitat gekennzeichnet? Z. B. Teilnehmer*innennummer	5-9, Tab.3
Konsistenz der Daten und Ergebnisse	30	Sind die vorgelegten Daten mit den Ergebnissen konsistent?	5-9
Schärfe/ Eindeutigkeit der Hauptthemen	31	Wurden die wichtigsten Themen in den Ergebnissen klar dargestellt?	5-9
Schärfe/Eindeutigkeit der Unterthemen	32	Gibt es eine Beschreibung diverser Fälle oder eine Diskussion/ Erörterung von Unterthemen?	5-9

Entwickelt von: Tong A, Sainsbury P, Craig J. Consolidated criteria for reporting qualitative research (COREQ): a 32-item checklist for interviews and focus groups. *International Journal for Quality in Health Care*. 2007. Volume 19, Number 6: pp. 349 – 357

*Hinweis: Diese Übersicht stellt eine eigenständige, nicht-offizielle Übersetzung der COREQ-Checkliste (Consolidated Criteria for Reporting Qualitative Research) dar. Die Übersetzung erfolgt ohne offizielle Validierung durch die Autor*innen oder das EQUATOR Network. Bei Unklarheiten oder für die Originalformulierungen wird die Konsultation der englischen Originalversion empfohlen.*

Anhang 2: Auszug Interview-Leitfaden für Fachpersonal der Rehazentren

Thema/inhaltliche Einleitung in das Projekt

Sie sind als Mitarbeiter*in dieser Reha-Einrichtung aktiv in dem ambulanten Rehaprogramm involviert und entsprechend übernehmen sie ja auch eine gewissen Rolle in dem Rehabilitationsprozess. Im Rahmen dieses Interviews möchte ich mich gerne mit Ihnen u.a. rundum die Teilnahme dieses Programm unterhalten.

Hauptfrage	Rückfrage	Memos
**Einstieg/ Icebreaker**		
Sie selbst sind Mitarbeiter*in des Rehabilitationszentrums und aktiv in das Reha-Programm involviert. Würden Sie mir zum Einstieg ein bisschen erzählen, wie so ein typischer Arbeitsalltag von Ihnen aussieht?	Was macht Ihre Arbeit im Rahmen des Programmes aus?	
**Hauptteil**		
**Thema: Reha-Programm**		
Nun ein wenig zum Reha-Programm an sich.<br>Wie sind Sie selbst zur Mitarbeit in dem Programm gekommen?		
Wie würden Sie das Rehaprogramm bewerten?	Welche Erfolgschancen sehen Sie in diesem Programm?	Haben Sie Beispiele für Erfolgschancen?<br>Was heißt in diesem Kontext „Erfolg“ für Sie?
Welche Ressourcen brauchen die KiJu/Eltern bei sich zu Hause bzw. innerhalb der Familie, um Gelerntes auch langfristig umzusetzen?	In welcher Form wird erfragt und sichergestellt, ob und wem die Ressourcen zur Verfügung stehen?	Ressourcen wären hier etwa ökonomische für gesünderes Essen oder Sport im Verein oder auch zeitliche, emotionale etc.
	Wie werden Sie dabei unterstützt?	
Welche Herausforderungen nehmen Sie als Mitarbeiter*in im Rahmen der Durchführung des Programmes wahr?	Wie begegnen Sie diesen Herausforderungen?	Herausforderungen jeglicher Art rund um die ambulante Reha (Beantragung, Durchführung, Übertragung in den Alltag, Nachsorge)
	Was müsste sich ändern, bzw. welche Möglichkeiten gäbe es, um bestehende Herausforderungen/ Hürden abzubauen?	
	Welche Herausforderungen gibt es in direktem Zusammenhang mit der Arbeit mit den KiJu und den Eltern?	

Mitarbeiter*innen & KiJu/Eltern		
Jetzt ein wenig weg von den Strukturen und mehr zu der Arbeit an sich. Was kommt Ihnen in den Kopf bei der Frage, was Ihnen an Ihrer Arbeit beim Reha-Programm Freude bereitet?<br>Und was bereitet Ihnen weniger Freude?	In Bezug auf:<br>• Wie nehmen Sie die Arbeit mit den Rehabilitand*innen wahr?<br>• Wie mit den Eltern?	
**Optimierung des Programms**		
Zu einem Programm gehört vermutlich immer auch Veränderung. Herangehensweisen werden eventuell überdacht oder es wird geschaut, wie Patient*innen noch anders unterstützt werden können.<br>Wenn Sie nun einmal spontan überlegen, was würden Sie sagen: Wie ließe sich das Programm noch erweitern bzw. verbessern?	• Welche wichtigen Aspekte fehlen, welche durch das Programm noch nicht abgedeckt werden?	
Was würden Sie an dem Programm verändern?	• Was könnte anders strukturiert sein bzw. am Konzept verändert werden?<br>• Welche Punkte müssten verändert werden, um die Patient*innen besser unterstützen zu können und z.B. auf individuelle Bedürfnisse der Rehabilitand*innen einzugehen?<br>• Welche Punkte müssten verändert werden, um die Eltern besser einzubeziehen? Oder vielleicht auch an manchen Stellen weniger?<br>• Was müsste verändert werden, um es mehr Familien und Kindern zu ermöglichen, am Programm teilzunehmen?	
**Ende**		
Nun sind wir schon am Ende des Interviews angelangt. Gibt es von Ihrer Seite denn noch Themen, die Sie gern noch ansprechen wollen, über die wir nicht gesprochen haben?		
Dann herzlichen Dank, dass Sie sich die Zeit genommen haben mit uns zu sprechen! Ich werde jetzt das Aufnahmegerät ausschalten.		

Anhang 3: Darstellung ergänzender Zitate

Ergänzende Zitate
**Ziele des ambulanten Rehaprogramms**
„Den Kindern und Eltern muss von Anfang an ziemlich klar sein, dass es nicht darum geht, in dieser Reha pro Kind 10 Kilo abzunehmen, was ja meist, meist eine Vorstellung ist, die die Kinder gerne hätten. Sondern es geht um die Umstellung, sie müssen Dinge anders machen, sie müssen ein anderes Verständnis für Körper bekommen, auch für den Umgang mit sich selbst und mit anderen."
„Natürlich ist es schön, wenn ein paar Gramm, ein paar Kilogramm purzeln, aber das ist für mich noch nicht mal so das Allerwichtigste."
**Erfolge des ambulanten Rehaprogramms**
„Also ein Erfolg für uns ist eigentlich, wenn in der Abschlussuntersuchung die Familien uns sagen, dass sie es geschafft haben, nachhaltig etwas in ihrem Alltag zu verändern. Aber von den Größen her können wir ja eigentlich nur das Gewicht erfassen und den BMI. Was wir aber noch machen ist ja auch ein Motoriktest, zum Beispiel in der Sporttherapie, wo es ja ganz häufig ist, dass die Kinder sich sportlich doch verbessert haben, gerade was den Ausdauer- und den Kraftbereich angeht und da ist es für uns natürlich schön, wenn die Familien uns dann in den Abschlussgesprächen erzählen, dass sich im Sportunterricht um eine Note verbessert wurde und es leichter fällt den Fahrradweg von zuhause bis zur Schule zu absolvieren. Solche Dinge sind für uns natürlich ein großer Erfolg."
**Herausforderungen des ambulanten Rehaprogramms**
Einfluss der Eltern im ambulanten Rehaprogramm
„Wo es auch hapert ne, also diese Veränderung auch zu machen oder, dass die Familien sich dafür Zeit nehmen (…). Also, dass die nicht sehen, dass alle mitmachen müssen, um da Veränderung zu machen und nicht: Das Kind kriegt jetzt DAS und WIR essen was anderes."
„Erstmal die große Baustelle Zeit. Also wie kriege ich meinen Alltag so strukturiert, dass Eltern und Kind irgendwo gemeinsam Zeit HABEN und das fällt natürlich dann meistens aufs Wochenende und dann hört man von den Eltern: ‚Ja, am Wochenende möchte ich mich ja auch mal auf die Couch legen'. Dann sage ich: ‚Okay, das verstehe ich voll und ganz, aber der Tag hat ja auch noch viel mehr Stunden, und dass man dann eben dort ansetzt und diese ganzen Sachen versucht ins Laufen zu bringen." <br>***Mangelnder Bekanntheitsgrad des ambulanten Rehaprogramms***

„Wobei wir so die Werbetrommel gerührt haben und trotzdem kommen sehr wenig Anfragen. Wir hatten eigentlich immer Gruppen in einer Stärke von acht, neun Kindern. Das haben wir jetzt zwei Mal schon nicht mehr geschafft, weil die Anfragen nicht da sind. Wir waren an den Schulen, wir waren bei den Kinderärzten, wir waren in den Krankenhäusern, wir waren beim Radiosender. Also es ist, oder sollte eigentlich bekannt sein.“
**Transfer in den Alltag**
„Im Moment ist es so: Die [KiJu] machen das Programm und dann ist es zu Ende. Und man weiß irgendwie, da wird sich nicht viel ändern. Man sollte das Programm vielleicht auf ein paar Jahre ausweiten, dass man vielleicht schafft eine Sportgruppe zu bilden und die Kinder kommen immer wieder. Dass es einfach bisschen langfristiger gemacht, länger geplant wird. Weil diese paar Monate reichen nicht aus, um das Problem zu lösen. Es ist ein guter Start, aber dann ist es auch wieder weg.“
